# Neutrophil‐Rich Infusion Site Reactions After Continuous Subcutaneous Application of Foslevodopa/Foscarbidopa

**DOI:** 10.1002/mds.30121

**Published:** 2025-01-11

**Authors:** David Weise, Sebastian Haferkamp

**Affiliations:** ^1^ Department of Neurology Asklepios Fachklinikum Stadtroda Stadtroda Germany; ^2^ Department of Neurology University of Leipzig Leipzig Germany; ^3^ Department of Dermatology University Hospital Regensburg Regensburg Germany

We read with great interest the article by Yoshihara et al.,^1^ which provides insight into histopathologic features of cutaneous side effects caused by continuous subcutaneous injection of foslevodopa/foscarbidopa. Using a similar approach, we analyzed skin biopsies from two female patients with Parkinson's disease (PD) who developed an inflammatory injection site reaction 11 and 13 weeks, respectively, after initiating subcutaneous treatment with foslevodopa/foscarbidopa. Notably, our histopathologic findings differ from those reported by Yoshihara et al., revealing a neutrophil‐rich inflammatory infiltrate.

## Patient 1, age 67 years, body mass index (BMI) 17.3 kg/m^2^

**FIG. 1:**
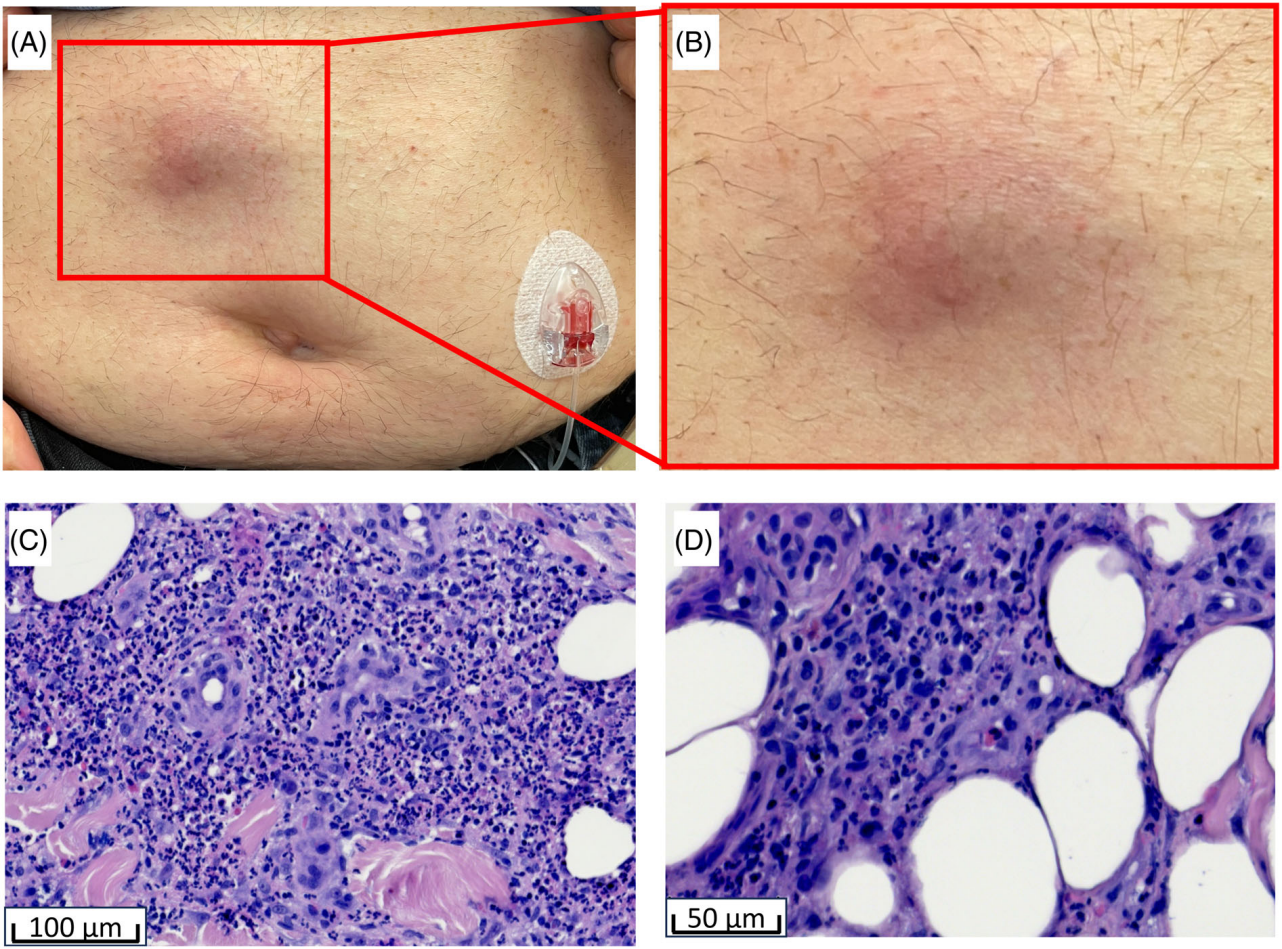
(A,B) Poorly demarked, dome‐shaped, erythematous swelling on the lower abdomen. (C,D) Neutrophil‐rich immune cell infiltrate in the lower dermis and subcutis. [Color figure can be viewed at wileyonlinelibrary.com]

Akinetic‐rigid type, disease duration 24 years, Hoen and Yahr scale (H&Y) 4 ON, 5 OFF with severe motor fluctuations and dyskinesia, optic hallucinations and PD dementia, previously treated with continuous subcutaneous apomorphine for 3 years, immediate change to foslevodopa/foscarbidopa due to not well‐controlled motor fluctuations and increasing optic hallucinations and delusion. Good improvement of motor fluctuations and dyskinesia. After 13 weeks of treatment (foslevodopa total dose 2592 mg, day rate 0.50 mL/hr, night rate 0.35 mL/hr, cannula change frequency [initially] 3 days) an oval, tender, poorly demarked, dome‐shaped, erythematous swelling was noted around the infusion site (Fig. 1A,B). Patient denied itching or pain.

## Patient 2, age 50 years, BMI 24.9 kg/m^2^

Akinetic‐rigid type, disease duration 15 years, H&Y 3 ON, 5 OFF with severe motor fluctuations and severe dyskinesia, previously treated with continuous subcutaneous apomorphine for 6 months (cessation due to insufficient improvement of fluctuations and persistent nausea), start of foslevodopa/foscarbidopa 8 months later with very good improvement of motor fluctuations and dyskinesia. She developed a painless, oval, poorly demarked, erythematous plaque measuring 5 cm in diameter after 11 weeks of treatment (foslevodopa total dose 2861 mg, day rate 0.52 mL/hr, night rate 0.45 mL/hr, cannula change frequency 2 days, relevant concomitant medication with opicapone 50 mg 1×/day).

Histopathologic examination of both cases revealed a patchy inflammatory infiltrate in the deep dermis extending into the subcutaneous tissue, composed primarily of neutrophils mixed with lymphocytes and a few eosinophils (Fig. 1C,D). In contrast to our findings, Yoshihara et al. described the adverse skin reactions as lymphocyte‐dominant inflammatory infiltrates in the adipose tissue. Interestingly, an eosinophil‐rich panniculitis has been observed in response to subcutaneously administered apomorphine,^2^ suggesting that the cellular components of immune responses to subcutaneous drug application may vary significantly. This notion is supported by the fact that a broad clinical spectrum of cutaneous side effects, including erythema, edema, cellulitis, panniculitis, subcutaneous nodule formation, and abscess formation, has been reported for both subcutaneous treatment regimens.^3^, ^4^ Infusion site reactions can be minimized by following best practices, including rotating injection sites, using a sterile injection technique, ensuring proper skincare and hygiene, monitoring for adverse reactions, and educating patients and their caregivers.^5^ However, further studies involving larger cohorts are needed to better understand the pathophysiology, identify risk factors, and explore potential prevention and treatment strategies of cutaneous side effects.

## Author Roles

Research Project: A. Conception and Design, B. Organization, C. Execution; (2) Statistical Analysis: A. Design, B. Execution, C. Review and Critique; (3) Manuscript Preparation: A. Writing of the First Draft, B. Review and Critique.

D.W.: 1A, 1C, 2B, 3A, 3B.

S.H.: 1A, 1C, 2B, 3A, 3B.

## Financial Disclosures

D.W. has received honoraria for advisory boards and speaker engagements from AbbVie, BIAL, Ever Pharma, and Stadapharm. S.H. has received honoraria for advisory boards and speaker engagements from AbbVie.

## Data Availability

The data that support the findings of this study are available from the corresponding author upon reasonable request.
